# Mousetap, a Novel Technique to Collect Uncontaminated Vitreous or Aqueous and Expand Usefulness of Mouse Models

**DOI:** 10.1038/s41598-018-24197-2

**Published:** 2018-04-23

**Authors:** Seth D. Fortmann, Valeria E. Lorenc, Jikui Shen, Sean F. Hackett, Peter A. Campochiaro

**Affiliations:** Departments of Ophthalmology and Neuroscience, Johns Hopkins University School of Medicine, Baltimore, Maryland USA

## Abstract

Vitreous or aqueous humour taps are widely used in patients or large animals with retinal diseases to monitor disease biomarkers, search for novel biomarkers, assess the integrity of the blood-retinal barrier, or perform pharmacokinetic or pharmacodynamics studies. Although there are many useful mouse models of retinal diseases, the small size of mouse eyes has precluded vitreous or aqueous taps. Herein we describe a novel technique, mousetap, which allows collection of vitreous or aqueous humour uncontaminated by blood or tissue surrounding the vitreous cavity. Mousetap was used to obtain vitreous samples from several mouse models of retinal vascular diseases and vitreous albumin measured by ELISA was highly reproducible among mice of the same model. The mean vitreous albumin concentration differed widely among control mice and mice of different models and correlated with fluorescein angiographic assessment of vascular leakage severity. Protein arrays showed increases in levels of several vasoactive proteins in the vitreous from mice with oxygen-induced ischemic retinopathy compared with age-matched controls; almost all of these proteins are increased in the vitreous of patients with the most common human ischemic retinopathy, proliferative diabetic retinopathy. Thus, mousetap facilitates the use of mice for studies previously reserved for large animal models and patients.

## Introduction

The eye is a relatively isolated compartment which contains the retina, a part of the central nervous system. The retina is affected by many prevalent vascular and neurodegenerative diseases, and development of treatments for these diseases is a high priority. In addition, therapeutic effects of anti-angiogenic, anti-permeability, or neurotrophic agents may predict effects in other tissues of the body; thus, the eye is often used as a model system. The retina lines the inner surface of the wall of the eye and borders the vitreous cavity filled with vitreous, a clear viscous fluid. Proteins secreted by the retina enter the vitreous and therefore levels of particular proteins in the vitreous are markers for function or dysfunction of the retina. Most of the proteins secreted into the vitreous diffuse into aqueous humour in the anterior chamber and exit the eye through the trabecular meshwork, the main outflow channel of the eye. The concentration of a protein in aqueous humor reflects its level in the vitreous and the retina^1,2^. Thus, analysis of aqueous or vitreous samples provides important information regarding the status of the retina. Vitreous samples obtained at the time of surgery for retinal diseases have provided insights into proteins involved in various diseases^3–5^. Aqueous humour samples can be obtained in the outpatient clinic allowing serial measurement of analytes which is extremely useful for investigating the molecular pathogenesis of disease and for pharmacokinetic and pharmacodynamic studies^6^.

Studying vitreous and aqueous samples in patients is particularly useful for exploring the molecular pathogenesis of diseases for which there are no good animal models, but for diseases for which animal models exist, it is also useful, because it provides a means of comparing molecular biomarkers in the model with those seen in the corresponding human disease. Once such validation is obtained, the value of the model is increased. Aqueous and vitreous samples are easily obtained from rabbits and monkeys which have relatively large eyes, but they are difficult to obtain from rodents. However, the ability to manipulate the genome of rodents has led to the generation of many rodent models that mimic key aspects of human diseases. In this study, we describe a novel way to obtain vitreous and aqueous samples in rodents and illustrate several ways in which those samples are useful.

## Results

### Mouse vitreous taps

**Figure 1:**
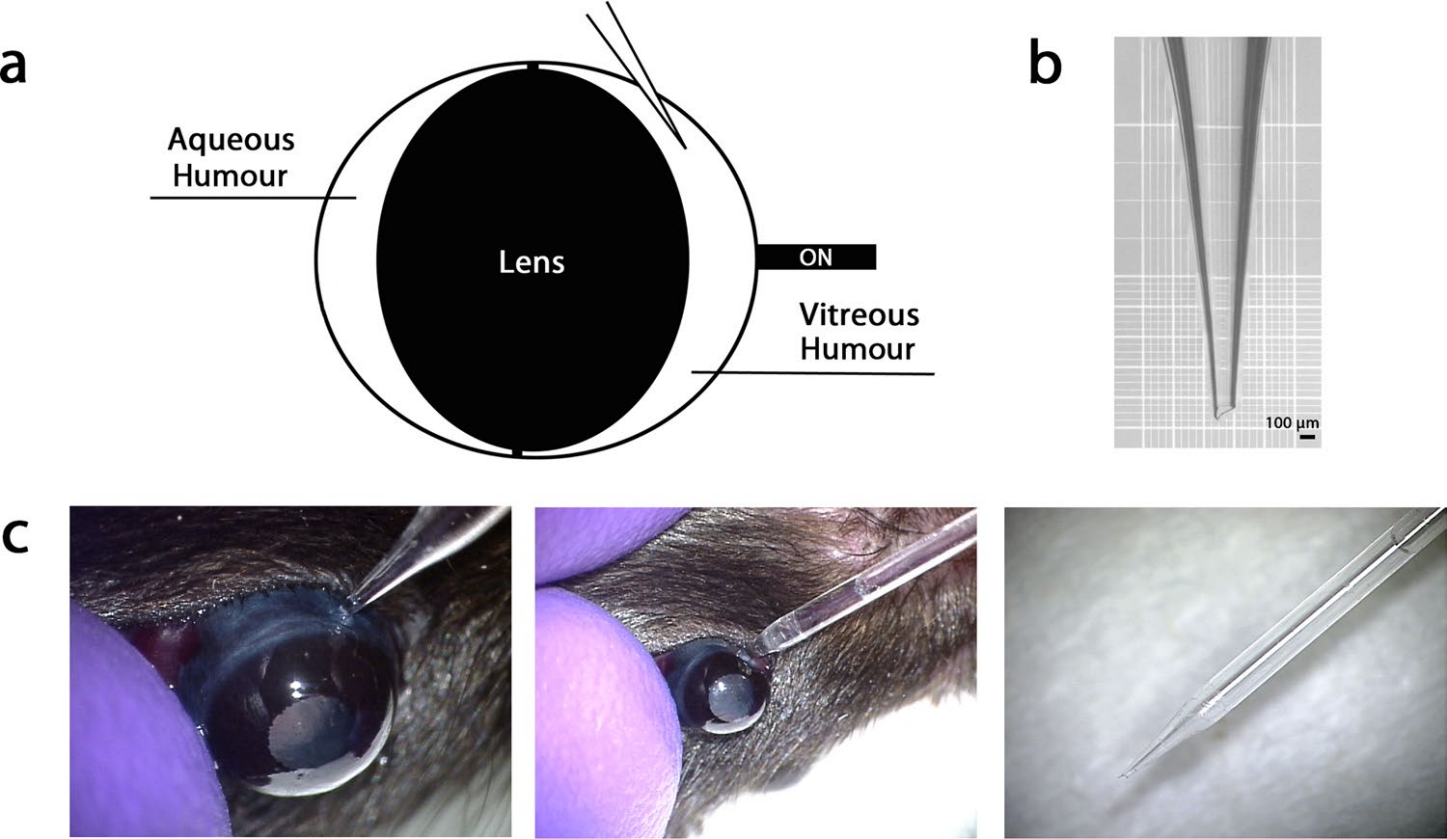
Mousetap technique. (**a**) Diagram of mouse eye showing aqueous humour anterior to the lens and vitreous humour posterior to the lens. The vitreous cavity is a narrow crescent that must be entered carefully to avoid the lens. The optic nerve (ON) exits from the posterior pole of the eye. (**b**) A pulled glass micropipette is broken to provide a sharp tip with an opening of about 100 µm. (**c**) To perform mousetap, the foot activated pump is set on aspirate, the eye is proptosed and held firm by the thumb applying gentle pressure 180° from the injection site, the micropipette is oriented to avoid the lens, the sclera is penetrated 2 mm posterior to the limbus (left panel), the foot petal is depressed, and vitreous is aspirated into the pipette (middle panel). Right panel shows the total amount of vitreous obtained, about 3.5–4 µl.

Vitreous taps in mice are challenging because of the small size of the eye and relatively large size of the lens which occupies the majority of the eye leaving only a thin crescent between the lens and retina for the vitreous (Fig. 1a). Even intravitreous injections, which are trivial and routine in humans, are difficult in mice because the imposing lens must be avoided and the small vitreous cavity can accommodate only a volume of 1 µl without reflux becoming a problem. Pulled glass micropipettes and pumps to eliminate finger movements facilitate intravitreous injections in mice by reducing the size of the instrument inserted between the lens and retina and by reducing manipulation and movement that could result in lens or retinal contact. It occurred to one of the authors (SDF) that a pump used for intravitreous injection could easily be converted into a vacuum allowing aspiration of vitreous if the opening in the pipette was large enough to accommodate the viscosity of the vitreous. Vitreous could not be aspirated through pulled glass micropipettes unless the tip was broken to provide an inner diameter of 100 µm and a corresponding outer diameter of 150 µm(Fig. 1b). The procedure for mouse vitreous taps is as follows. A micropipette puller is used to pull the tips of standard glass pipettes. After cooling, the tapered tip of the micropipette is grasped with forceps and broken (supplemental video). Mice are anesthetized, the eye is proptosed in front of the eye lids, and the tip is inserted through the sclera 2 mm posterior to the limbus (Fig. 1c, left). Suction is activated by stepping on the foot petal with the pump in reverse mode and vitreous is slowly aspirated (Fig. 1c, middle) allowing collection of as much as 3.5–4 µl in 4–6 weeks old mice (Fig. 1c, right; supplemental video). The mice are then euthanized and although eye removal and retinal dissection are more challenging than normal because the eye is soft, the retina can be dissected allowing comparative studies on vitreous and retina.

### Mouse aqueous taps

A similar procedure can be used to obtain undiluted aqueous from mice. This is a terminal procedure unlike aqueous taps in humans which are minimally invasive and can be repeated many times. For aqueous taps, pulled glass micropipettes require a slightly narrower tip with an inner diameter of 80 µm and a corresponding outer diameter of 100 µm (Supplemental Fig. 1a). Unlike aqueous taps in humans or large animals, the cornea is not penetrated at the limbus, but rather the sharp tip of the broken pulled glass micropipette is inserted near the center of the cornea where separation from the lens is greatest (Supplemental Fig. 1b). As opposed to mouse vitreous taps, no forced aspiration is required because the viscosity of the aqueous is low and the small differential between intraocular and atmospheric pressure is sufficient to cause the aqueous to spontaneously enter the pipette (Supplemental Fig. 1b, second and third columns; supplemental video).

**Figure 2:**
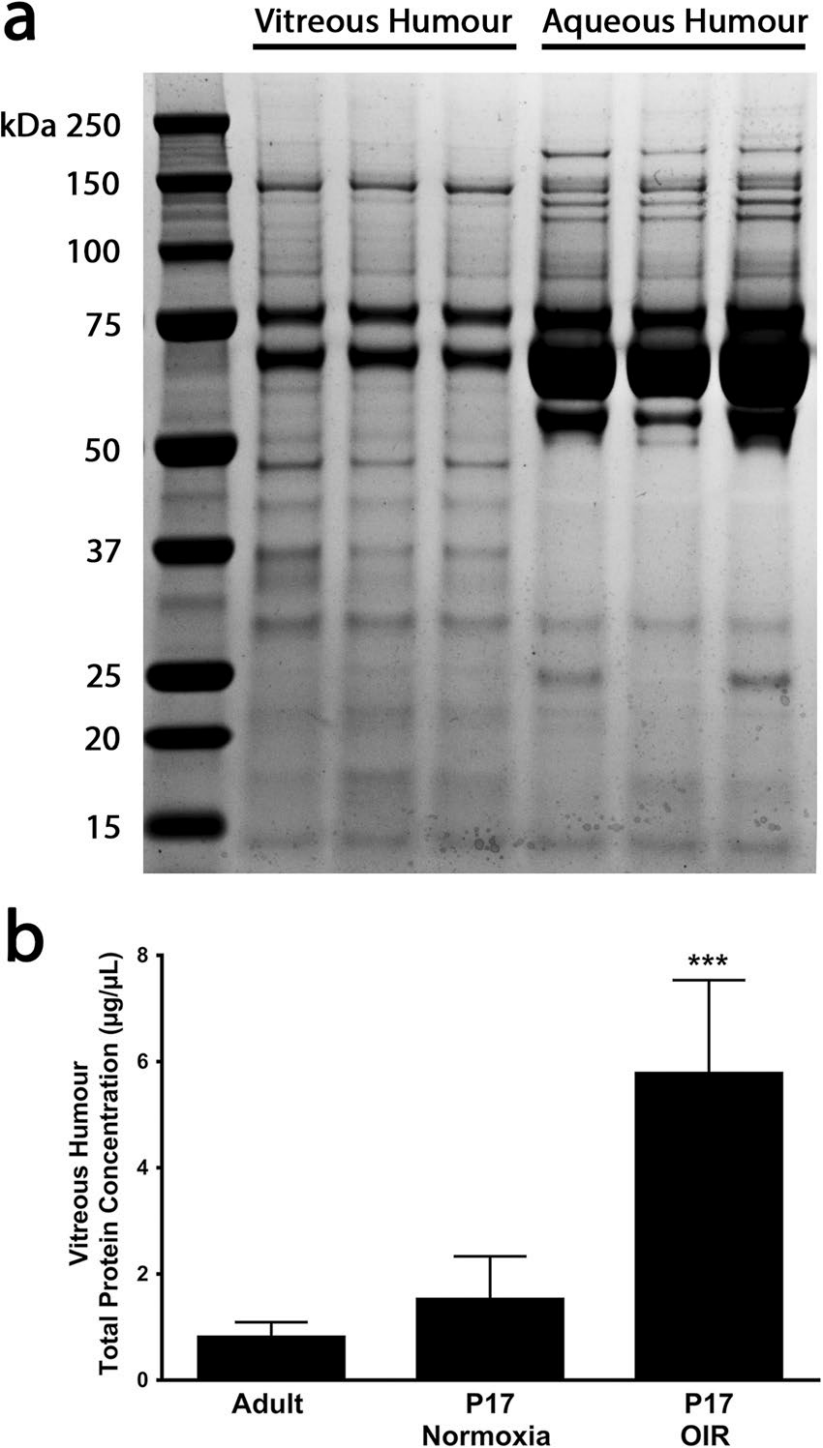
Mouse vitreous humour and aqueous humour proteomes. (**a**) SDS-PAGE and Coomassie blue staining of vitreous humour proteome versus aqueous humour proteome. The 3 lanes labeled vitreous humour are independent vitreous samples from 3 different mice and the 3 lanes labeled aqueous humor are aqueous samples from 3 different mice. (**b**) Bars (n = 5 for each) show mean ( ± SEM) total protein concentration in vitreous humour from eyes of normal adult mice, P17 mice raise in room air (normoxia), and P17 mice with oxygen-induced ischemic retinopathy (OIR). ***p < 0.001 by ANOVA with Dunnett’s correction for multiple comparisons.

Multiple independent samples verified that the proteome of aqueous is very different from that of vitreous indicating that these are distinct fluids (Fig. 2a). In adult mice and postnatal day (P) 17 juvenile mice, the mean protein concentration of the aspirated vitreous was less than 2 µg/µl, but in P17 mice with oxygen-induced ischemic retinopathy (OIR)^7^ in which there is extensive retinal neovascularization (NV) and severe breakdown of the blood-retinal barrier, the mean protein concentration of the aspirated vitreous was significantly greater, approximately 6 µg/µl (Fig. 2b).

### Mousetap facilitates quantitative assessment of retinal vascular leakage

**Figure 3:**
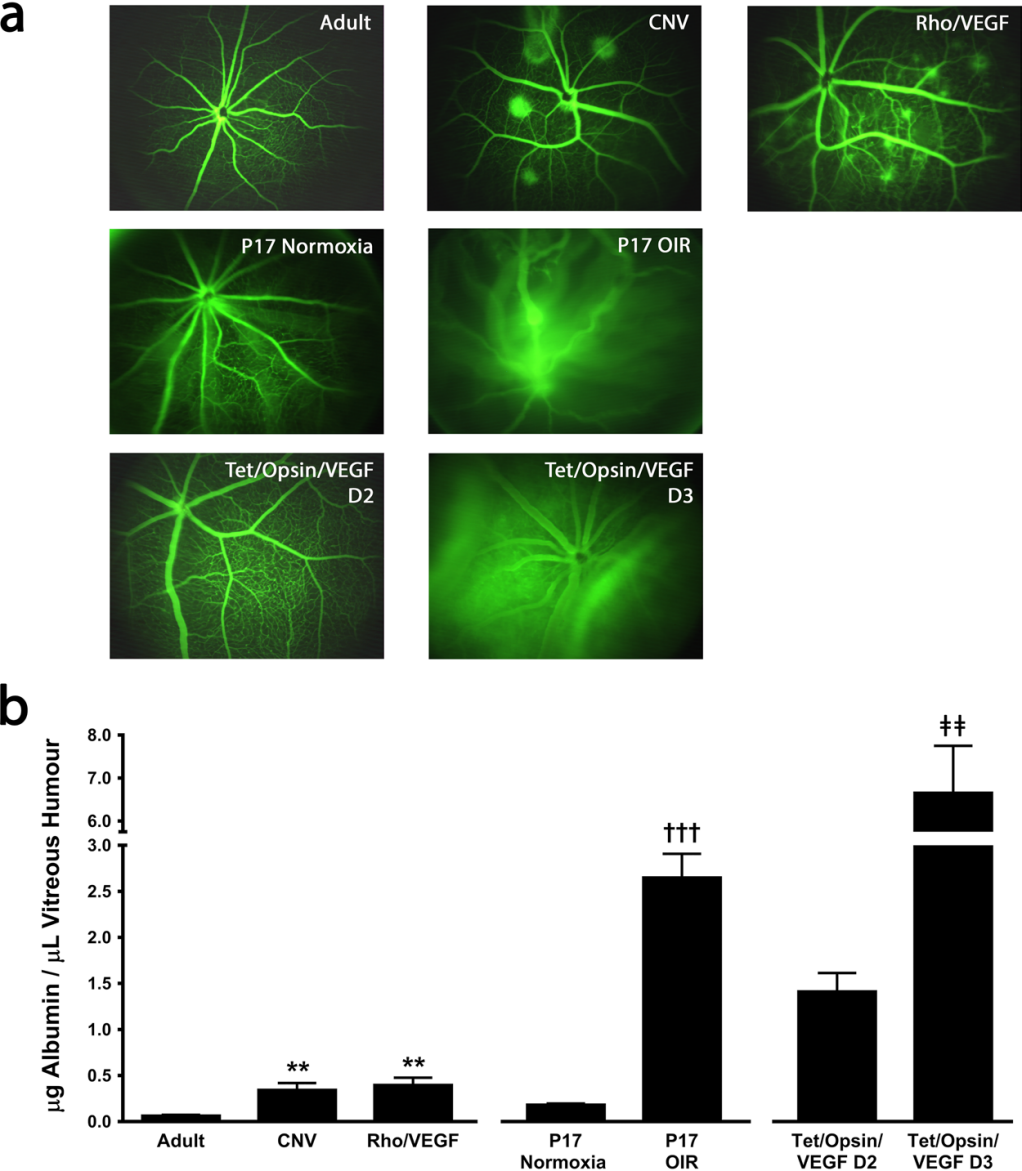
Quantification of blood-retinal barrier breakdown in mouse models by measurement of albumin concentration in vitreous humour. (**a**) Fluorescein angiogram of a normal adult mouse (row 1, column1), mouse with choroidal neovascularization (CNV) due to laser-induced rupture of Bruch’s membrane (row 1, column 2), transgenic mouse in which the *rhodopsin* promoter drives expression of VEGF in photoreceptors (Rho/VEGF; row 1, column 3), P17 mouse raised in room air (P17 normoxia; row 2, column 1), P17 mouse with oxygen-induced ischemic retinopathy (P17 OIR; row 2, column 2), and double transgenic mouse with doxycycline-inducible expression of VEGF in photoreceptors treated with 2 mg/ml of doxycycline in drinking water for 2 days (Tet/Opsin/VEGF D2; row 3, column 1) or 3 days (Tet/Opsin/VEGF D3; row 3, column 2). (**b**) Bars (n = at least 5) show mean ( ± SEM) albumin concentration in vitreous humour measured by ELISA for each of the mouse models shown in (a). **p < 0.01 compared with normal adult by ANOVA with Dunnett’s correction; ^†††^p < 0.001 compared with P17 normoxia by Student’s t-test; ^ǂǂ^p < 0.01 compared with Tet/Opsin/VEGF D2 by Student’s t-test.

Leakage from pre-existent retinal vessels or from NV is a major source of reduced vision in patients with retinal/choroidal vascular disease; therefore, procedures to assess leakage are used in clinical care and clinical trials and it is important to test the efficacy of new anti-permeability agents in validated mouse models. Fluorescein angiography is a good technique to detect and localize leakage, but it is not quantitative. In adult mice, large retinal vessels are narrow with sharp borders and small vessels are regular and somewhat indistinct in a low magnification view (Fig. 3a, top row, first column). In mice with choroidal NV due to laser-induced rupture of Bruch’s membrane^8^, which corresponds to type 2 choroidal NV (originates from the choroid and penetrates through Bruch’s membrane and the retinal pigmented epithelium (RPE) into the subretinal space) in patients with neovascular age-related macular degeneration, there are irregular spots of hyperfluorescence with fuzzy borders beneath retinal vessels which have sharp borders and appear distinct from the choroidal NV (Fig. 3a, top row, second column). Transgenic mice in which the *rhodopsin* promoter drives expression of VEGF in photoreceptors (*rho/VEGF* mice) develop type 3 choroidal NV which originates from the deep capillary bed of the retina, grows through the photoreceptor layer, and forms networks of new vessels in the subretinal space^9,10^. Fluorescein angiography of *rho/VEGF* mice shows hyperfluorescent spots with indistinct borders that appear deep to large retinal vessels which appear distinct, but connect with small retinal vessels which is difficult to see on the low magnification images of a fluorescein angiogram (Fig. 3a, top row, third column) but is seen in magnified views of a retinal whole mount incubated with a FITC-labeled lectin that selectively stains vascular cells (see Fig. 2a in Lui *et al*.^11^). Retinal vascular development starts at postnatal day (P) 0 and is completed by P28. Fluorescein angiography of a P17 mouse raised in room air (P17 normoxia, Fig. 3a, second row, first column) shows retinal vessels with fuzzy borders indicating that the blood-retinal barrier is not completely formed at this stage of development. Mice with OIR are placed in 70% oxygen at P7 which results in down-regulation of VEGF and regression of newly formed retinal vessels and when returned to room air at P12, there are areas of ischemic retina resulting in upregulation of VEGF and other hypoxia-regulated gene products followed by growth of retinal NV^7^. Fluorescein angiography of a P17 mouse with OIR shows dilated retinal vessels with fuzzy borders and a diffuse green haze indicating substantial extravascular fluorescein (Fig. 3a second row, second column). After the administration of 2 mg/ml doxycycline in drinking water, double transgenic *Tet/opsin/VEGF* mice express VEGF in photoreceptors which increases over time and after 3 days VEGF levels are more than 30-fold higher than those seen in *rho/VEGF* mice^12^. Leakage from retinal vessels also increases over time and 4 days after starting doxycycline, it is so severe that most *Tet/opsin/VEGF* mice develop total exudative retinal detachment. Fluorescein angiography 2 days after starting doxycycline shows dilated vessels allowing small vessels to be very well-seen (Fig. 3a, third row, first column), but 3 days after starting doxycycline small vessels are obscured by severe leakage and diffuse green haze from extravascular fluorescein (Fig. 3a, third row, second column).

Measurement of albumin in vitreous samples obtained by mousetap provides precise quantification of the amount of vascular leakage in each of the models describe above. Compared with normal adult mice, the mean vitreous concentration of albumin is significantly greater in adult mice with choroidal NV at 5 rupture sites in Bruch’s membrane or adult *rho/VEGF* mice (Fig. 3b). Compared with P17 mice raised in room air, mean vitreous albumin concentration was significantly greater in P17 mice with OIR. Mean vitreous albumin concentration was significantly greater in samples obtained from *Tet/opsin/VEGF* mice 3 days after onset of doxycycline compared with samples obtained 2 days after starting doxycycline.

### Mousetap provides a useful way to compare vitreous levels of proteins in normal and diseased eyes

**Figure 4:**
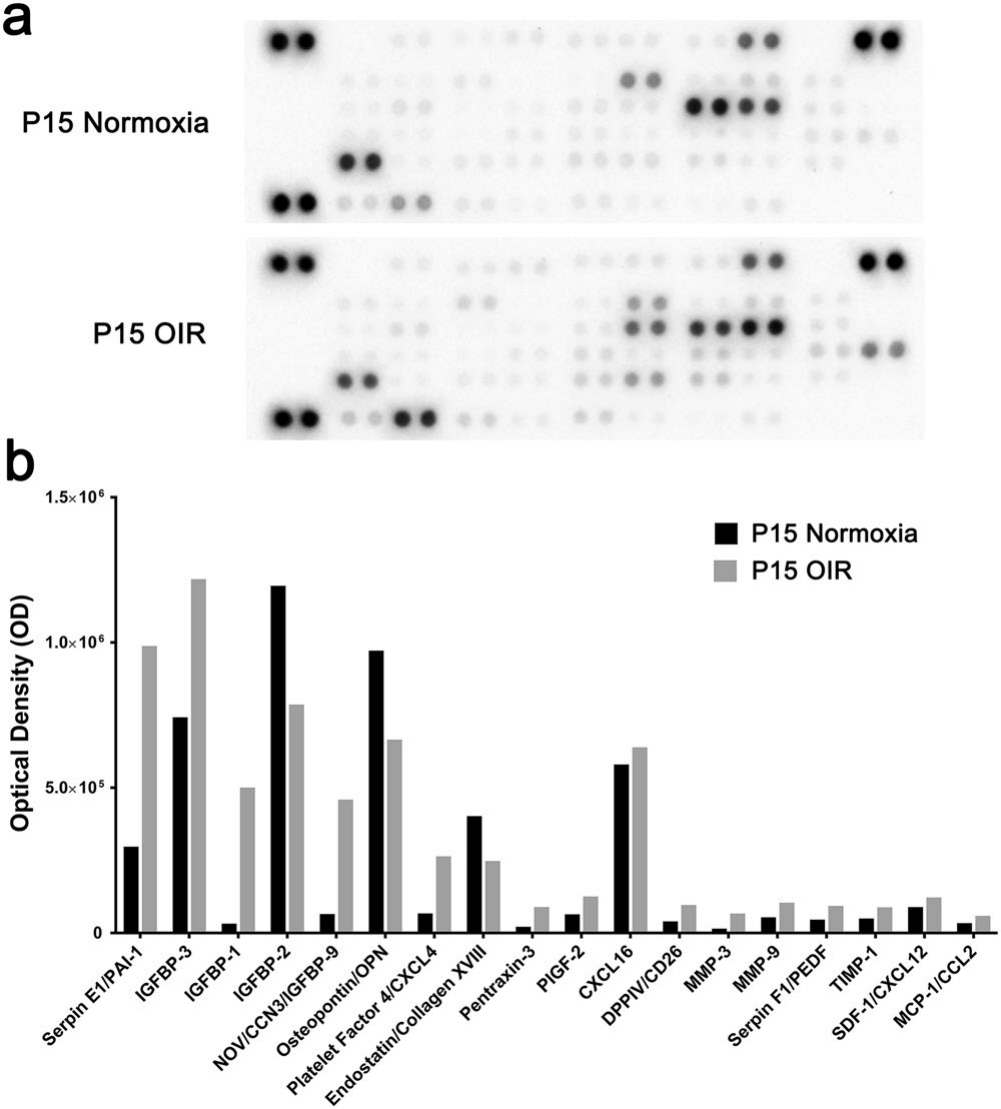
Comparisons of angiogenesis-related proteins in vitreous humour from eyes of normal P15 mice and P15 mice with oxygen-induced ischemic retinopathy (OIR). Pooled vitreous humour samples of equivalent volume from P15 mice raise in room air and P15 mice with OIR (20 mice, 40 eyes for each condition) were run in angiogenesis protein arrays. (**a**) Images of membranes from arrays. (**b**) Each bar is the average optical density from duplicate wells for detectable proteins.

At P15, twenty mice with OIR and twenty control mice raised in standard room air conditions had vitreous samples obtained from each eye by mousetap. The 40 samples for each group were pooled and 120 µl of the pooled samples were assayed in angiogenesis protein arrays. Compared with vitreous from controls, that from OIR mice showed substantial increases in plasminogen activator inhibitor-1 (PAI-1), insulin-like growth factor binding protein-3 (IGFBP-3), IGFBP-1, IGFBP-9, platelet factor 4 (PF4), pentraxin-3 (PTX3), placental growth factor-2 (PlGF-2), dipeptidyl peptidase-4 (DPP-IV), matrix metalloproteinase-3 (MMP-3), MMP-9, pigment epithelial-derived factor (PEDF), tissue inhibitor of metalloproteinases-1 (TIMP-1), stromal-derived factor-1 (SDF-1), and macrophage colony stimulating factor-1 (MCP-1) (Fig. 4a and b).

## Discussion

The relative isolation of the eye from the rest of the body provides therapeutic benefits. One benefit is the ability to provide local administration of drugs to the eye. Intraocular injection of VEGF-neutralizing proteins is a breakthrough treatment for neovascular age-related macular degeneration, diabetic macular edema, and retinal vein occlusion^13^. Systemic suppression of VEGF has adverse effects that are tolerated in the treatment of life-threatening malignancies, but cannot be tolerated in patients with ocular diseases^14^. Intraocular injection of anti-VEGF agents allows usage of small amounts of these agents that slowly exit from the eye resulting in minimal systemic exposure.

Relatively isolated fluid-filled spaces, the vitreous cavity filled with vitreous and the anterior chamber filled with aqueous, can be accessed to obtain vitreous or aqueous samples. These samples provide diagnostic benefits including the ability to measure proteins such as VEGF that have been implicated as critical in the pathogenesis and progression of diseases^6^. Baseline aqueous levels of VEGF predict visual outcomes in patients with retinal vein occlusion treated with intraocular injections of a VEGF-neutralizing protein^15^. The ability to obtain serial aqueous samples from patients after intraocular injection of a small polymer cylinder that slowly releases fluocinolone acetonide allowed precise pharmacodynamic analysis and documentation that therapeutic levels are present in the eye for 3 years^16,17^. Likewise, serial measurements of transgene levels from aqueous samples has provided the first data documenting the level and duration of transgene expression after ocular gene therapy in patients^18,19^.

In this study we have described a novel technique to obtain vitreous and aqueous samples in mice and have illustrated types of investigations that these samples facilitate. Quantification of alterations in retinal vascular permeability and the effect of therapeutic agents to suppress excessive permeability is a very useful application. A common approach is to inject an exogenous tracer into the systemic circulation and then measure levels in the retina. This requires injection of the exact same amount of tracer in each animal which is generally assumed to be the case, but is rarely verified. Despite diligent attempts to inject the same amount of [^3^H]mannitol in different mice, systemic levels of [^3^H]mannitol varied, and it was necessary to account for differences in systemic levels among mice by calculating the retina:renal and retina:lung [^3^H]mannitol leakage ratios^20^. Another approach is to use serum albumin as an endogenous marker for extravascular leakage^21,22^. Immunohistochemistry for serum albumin provides localization and qualitative assessment of leakage. It is possible to measure albumin concentration in retinal homogenates by ELISA, but it is necessary to perfuse the mouse through the left ventricle with PBS to wash out all intravascular albumin prior to isolation of the retina. Since proteins that leak into the retina diffuse into the vitreous and because the vitreous is avascular, measurement of albumin in vitreous is much simpler and eliminates potential confounding factors encountered with measurements in retinal homogenates. Measurement of albumin levels in mouse models of ocular NV were consistent with qualitative assessments of vascular leakage severity by fluorescein angiography and were highly reproducible within groups of mice of the same model (Fig. 3). This suggests that quantitative assessment of retinal vascular leakage by measurement of albumin concentration in vitreous is a suitable approach to assess the efficacy of anti-permeability agents.

Experiments aimed at identifying proteins contributing to pathologic effects in the retina are another useful application for aqueous and vitreous samples. Almost all of the proteins that were substantially higher in the vitreous of mice with OIR versus age-matched controls have been previously demonstrated to be increased in the vitreous of patients with PDR compared with controls, including PAI-1^23^, IGFBPs^24–26^, PTX3^27^, PlGFs^28,29^, MMPs^5,30,31^, TIMP-1^32^, SDF-1^33^, and MCP-1^34,35^. These molecular correlations support the anatomic correlations of retinal nonperfusion and retinal NV, indicating that the OIR mouse model recapitulates critical aspects of PDR and that vitreous samples obtained from OIR mice are likely to be useful for investigations of the molecular pathogenesis of PDR and other human ischemic retinopathies.

In conclusion, the procedures to obtain vitreous and aqueous samples from mice described herein are useful tools that facilitate quantitative assessment of the blood-retinal barrier, pharmacokinetic and pharmacodynamics studies, and studies exploring the molecular pathogenesis of retinal diseases.

## Methods

### Mice

Mice were treated in accordance with the ARVO Statement for Use of Animals in Ophthalmic and Vision Research and protocols were reviewed and approved by the Johns Hopkins University Animal Care and Use Committee. All transgenic mice were in a C57BL/6 background and were genotyped by PCR for validation of transgenes. Wild type C57BL/6 mice were purchased from Charles River (Frederick, MD).

### Mouse model of OIR

Ischemic retinopathy was induced in neonatal mice as previously described^7^. In brief, at postnatal day 7 (P7), wild-type mothers and pups were placed in hyperoxia chambers (75 ± 3% O_2_) for 5 days. At P12, the mice were returned to room air and ischemic retinopathy was allowed to develop until P15 or P17 as indicated.

### Laser-induced choroidal NV in mice

Type 2 choroidal NV was induced in adult wild type mice by laser photocoagulation-induced rupture of Bruch’s membrane as previously described^8^. In brief, 4–6 week old mice were anesthetized, pupils were dilated with 1% tropicamide (Alcon Labs, Inc., Fort Worth, TX), and Bruch’s membrane was ruptured at 3, 5, 8, 10, and 12 o’clock positions 1 mm from the optic nerve with 532 nm diode laser photocoagulation (75 µm spot size, 0.1 s duration, 130 mW) using the slit lamp delivery system of an OcuLight GL Photocoagulator (Iridex, Mountain View, CA) and a cover slide as a contact lens.

### Transgenic mice with expression of VEGF in photoreceptors

Transgenic mice in which the *rhodopsin* promoter drives expression of VEGF_165_ in photoreceptors (*rho/VEGF* mice) develop type 3 choroidal NV that sprouts from the deep capillary bed of the retina and grows into the subretinal space^9,10^. *Rho/VEGF* mice and age-matched controls had fluorescein angiography at P30 and then vitreous samples were obtained by mousetap and albumin concentrations were measured. Double transgenic mice with inducible expression of VEGF_165_ in photoreceptors (*Tet/Opsin/VEGF* mice) develop severe leakage from retinal vessels resulting in exudative retinal detachment 4 days after initiation of 2 mg/mL doxycycline in drinking water^12^. Mousetap was done to obtain vitreous samples 2 or 3 days after initiation of doxycycline and albumin concentrations were measured.

### Mousetap

Mouse vitreous humour collection was done using the Harvard Pump Microinjection System (Harvard Apparatus, Holliston, MA). A Flaming/Brown Micropipette Puller (model P-97, Sutter Instruments, Novato, CA) was used to pull the tips of standard glass pipettes (100 mm in length with a 1 mm outer diameter and 0.75 mm inner diameter). The instrument settings were heat 280, pull 50, velocity 200, and delay 100. The micropipette tip was broken at an angle to provide a sharp tip with an inner diameter of 100 µm (outer diameter 150 µm), and was inserted at a 45° angle into the vitreous cavity 2 mm posterior to the limbus. The foot pedal was activated to slowly aspirate 3.5 µL of undiluted vitreous humour. Samples were immediately placed on ice, and the needle was flushed with sterile PBS in between collections. Samples were centrifuged at 6,000 rpm for 30 seconds and stored at −80 °C.

### Mouse aqueous humour collection

Mouse aqueous humour was collected using the Harvard Pump Microinjection System and pulled glass micropipettes. The micropipette tip was broken at an angle to provide a sharp tip with an inner diameter of 80 µm (outer diameter 100 µm) and was inserted through the center of the cornea into the anterior chamber. Aqueous humour spontaneously entered the pipette allowing collection of 4–5 µL of undiluted aqueous humour. Samples were immediately placed on ice and the needle was flushed with sterile PBS in between collections. Samples were centrifuged at 6,000 rpm for 30 seconds and stored at −80 °C.

### Measurement of total protein and separation of proteins by PAGE

Total protein concentration was measured using Bradford assay (Bio-Rad, Hercules, CA) for vitreous samples from adult wild-type mice, juvenile P17 normoxic mice, and P17 OIR mice. Proteins in aqueous and vitreous samples (10 µl) were incubated with Bolt LDS Sample Buffer and DTT (25 µM) (Thermo Fisher Scientific, Waltham, MA) at 95 °C for 5 minutes. Samples were separated on a NuPAGE 4–12% Bis-Tris gel under reducing conditions using MES SDS running buffer. Following electrophoresis, the gel was stained with Coomassie Blue R-250 (Bio-Rad) for 3 hours and visualized using the ChemiDoc XRS System (Bio-Rad).

### Fluorescein angiography

Adult wild-type mice, P17 normoxic mice, P17 mice with OIR, adult wild type mice with choroidal NV, P30 *rho/VEGF* mice, Tet/Opsin/VEGF mice at 2 or 3 days after addition of doxycycline to drinking water (2 mg/ml) were anesthetized, pupils were dilated with 1% tropicamide, and fundus photographs were obtained with a Micron III Retinal Imaging Microscope (Phoenix Research Laboratories Inc., Pleasanton, CA) before and at several time points after intraperitoneal injection of 50 µL of 25% fluorescein (AK-Fluor; Lake Forest, IL).

### Measurement of albumin in vitreous samples

Vitreous humour was collected from the eyes of adult wild-type mice, P17 normoxic mice, P17 mice with OIR, adult wild type mice with choroidal NV, P30 *rho/VEGF* mice, Tet/Opsin/VEGF mice at 2 or 3 days after addition of doxycycline to drinking water (2 mg/ml). Using the manufacturer’s instructions, a mouse albumin ELISA kit (ab108791; Abcam, Cambridge, MA) was used to measure albumin levels in 1 µL of diluted vitreous humour and albumin samples for standard curve generation. The plate was read at 450 nm and 570 nm.

### Angiogenic protein array of vitreous samples from P15 control and OIR mice

At P15, vitreous humour was collected from the eyes of control mice and mice with OIR. Using the manufacturer’s instructions, a mouse angiogenesis array kit (ARY015; R&D Systems, Inc, Minneapolis, MN) was used to compare 53 different angiogenesis-related proteins. Briefly, the prepared samples were incubated with the membrane overnight at 4 °C on a rotating platform. The membranes were then washed with buffer, incubated with Streptavidin-HRP conjugate and chemi reagent mix, and imaged together using the ChemiDoc XRS System (Bio-Rad). Images were analyzed together using the Bio-Rad imaging software and background corrected optical density was quantified for each spot. A difference in OD ≥ 20,000 was used to select proteins that were differentially increased or decreased in OIR versus control vitreous.

### Statistics

Statistical comparisons between multiple groups were done using one-way ANOVA with Dunnett’s correction and comparisons between two groups were done with Student’s t-test.

### Data availability

All data are available by contacting the corresponding author.

## Electronic supplementary material

Mouse Vitreous and Aqueous Taps Video

Supplemental Figure 1
