# Supplementary material for: Mousetap, a Novel Technique to Collect Uncontaminated Vitreous or Aqueous and Expand Usefulness of Mouse Models
Source: Sci Rep. 2018 Apr 23;8:6371. doi: 10.1038/s41598-018-24197-2 (PMC5913258; doi:10.1038/s41598-018-24197-2)
Supplement: Supplementary file 2 — Supplemental Figure 1 [file 41598_2018_24197_MOESM2_ESM.pdf]

Mousetap, a Novel Technique to Collect Uncontaminated Vitreous or Aqueous and Expand Usefulness of Mouse Models

Seth D. Fortmann†, Valeria E. Lorenc†, Jikui Shen, Sean F. Hackett, Peter A. Campochiaro*

Departments of Ophthalmology and Neuroscience
Johns Hopkins University School of Medicine
Baltimore, Maryland

Supplemental Figure 1

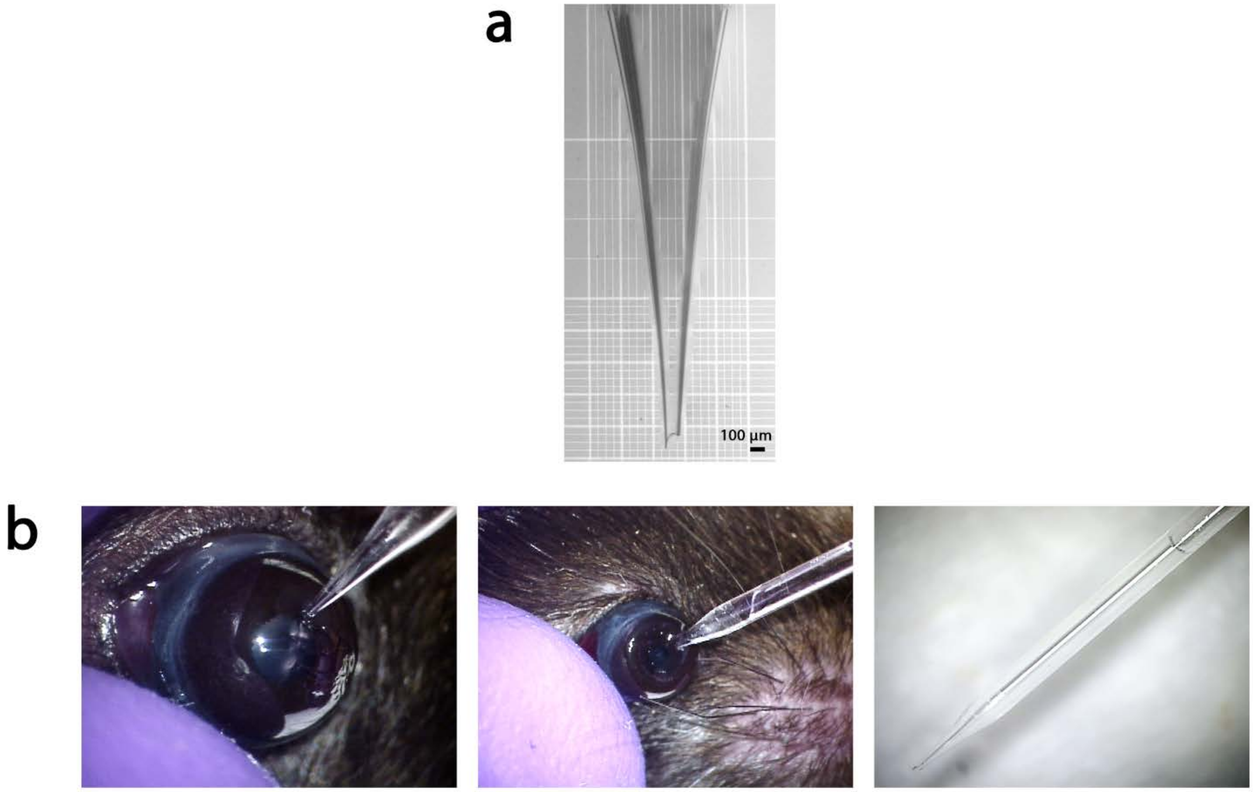

Supplemental Figure 1. Mouse aqueous humour tap technique. (a) Cleaved glass micropipette with an opening of about 80 µm. (b) Tip of the micropipette is positioned near the center of the cornea (left). Pressure is applied to puncture the cornea resulting in spontaneous entry of aqueous humor into the micropipette (middle) which contains about 4-5 µl at the end of the procedure (right).
